# Ignorance culture and eating disorders: lived experience analysis of alarms being ignored

**DOI:** 10.1192/bjb.2025.10199

**Published:** 2026-08

**Authors:** James Downs

**Affiliations:** Independent Researcher and Patient, Cardiff, UK

**Keywords:** Mental health services, psychological treatments, service development, patients and service users, feeding or eating disorders

## Abstract

**Summary** Despite co-produced guidelines and actions recommended by statutory bodies, there has been a persistent lack of progress in improving the quality of healthcare for those with eating disorders in the UK. Drawing on multiple evidence sources, including lived experience, the author analyses reasons for this. The concept of an ‘ignorance culture’ is theorised as a key barrier, defined as cultural practices that uphold systemic failures by ignoring concerns that harm clinicians, patients, caregivers and wider society. A shift towards a ‘responsibility culture’ is proposed, with recommendations aimed at creating greater accountability, shared learning, transparency and reflexivity. Prioritising cultural change is central to improving the quality of care for everyone affected by eating disorders.

When healthcare systems become unsafe, patients, carers and clinicians rely on feedback mechanisms to raise concerns and demand reform. However, as demonstrated by the 2017 Parliamentary Health Service Ombudsman (PHSO) report *Ignoring the Alarms: How NHS Eating Disorder Services are Failing Patients*, these processes often fail to be effective. Prompted by the preventable death of Averil Hart in 2012, the report revealed pervasive failures in National Health Service (NHS) eating disorder services, including insufficient training, poor care coordination and inequities in service provision.^1^ The Ombudsman emphasised in particular how Averil’s father, Nic, had raised concerns that had been continuously ignored.

Despite repeated calls for action, meaningful reforms to eating disorder care remain elusive. Hopes for transformation were briefly kindled in 2018 when NHS England convened a Delivery Group to implement the PHSO’s recommendations, of which I was a member. However, by the time the group disbanded in 2023, none of the recommendations had been fully implemented.^2,3^

Having lived with a severe eating disorder and multiple co-occurring conditions for over 20 years, I have felt the impact of the NHS continually ‘failing patients’ first-hand. In recent years, I have required numerous hospital admissions for acute illness, where I have been met with a near-total lack of awareness regarding treatment guidelines, leading to significant medical deterioration. Safeguarding concerns raised by other professionals were not acted upon by the eating disorder service overseeing my care, who also refused my requests to see their dietician. This was despite being informed by a gastroenterologist that I required a permanent liquid diet. The treatment available to me was either one session per week with the eating disorder team or an out-of-area admission to a specialist unit, which was offered with minimal planning and at short notice. I was denied admission to my local unit, even though a Care Planning Approach meeting recommended this as a reasonable adjustment. Throughout my treatment, I never met my named consultant, which only deepened my sense of neglect and lack of confidence in the healthcare system.

Although my experiences are anecdotal, the fact that such experiences can occur *at all* illustrates a system that is capable of failing patients at a structural level. These examples indicate a routine failure to implement statutory guidelines, including those for commissioners and providers of adult community eating disorder services^4^ and for treating medical emergencies.^5^ Moreover, when I raised concerns about my care, I encountered similar dismissals noted in the PHSO report. My dissatisfaction was attributed to my illness rather than the systemic problems that allowed for substandard treatment. I was labelled ‘demanding’ rather than being seen as a motivated patient who had fought hard to access any care at all. I do not deny the psychological challenges I have faced when trying to navigate services when severely unwell. However, I believe that explanations that interpreted the difficulties of my treatment entirely through the lens of my personal psychopathology served to deflect responsibility from my treatment providers, ignoring that the best care I was offered was a form of managed decline.

## The current study

Although well-documented resource constraints and limited training are known factors contributing to institutional inertia, the subject of organisational culture remains under-explored in the context of eating disorder services. In this paper, I draw on my personal experience alongside relevant theoretical frameworks and research to theorise that the enduring failures of NHS eating disorder services are being maintained by an organisational culture that is unwilling to take responsibility for addressing them.

## Method

### Terminology

I use the term ‘ignorance culture’ to describe the prevailing organisational culture in NHS eating disorder services, characterised by systems and practices that suppress patient feedback, silence dissenting voices and ensure that organisations resist the scrutiny required to bring about change. The phrase aims to capture the role of wilful blindness in shielding healthcare professionals and policymakers from accountability and moral injury,^6^ allowing harmful practices to persist unchecked. Patients and their caregivers, often acutely aware of the system’s failures, may be left to bear the brunt of these failings, with their warnings met with silence, deflection, or even blame.^7–9^

As well as being descriptive, the term ‘ignorance culture’ is used here as a rhetorical device for uncovering and critically analysing the cultural phenomena that sustain failures in eating disorder services across individual, interpersonal and organisational levels. Throughout, I take care to remain reflexive, resist assigning blame and focus on the pressing need to create safer, more equitable systems of care for the benefit of patients, caregivers and clinicians alike.

### Justification for theoretical framework

Central to this analysis is an examination of how discourse, power and organisational culture intersect to sustain long-standing failures within NHS eating disorder services. The theoretical frameworks I have chosen have been selected for their specific utility in interrogating the mechanisms by which responsibility is deflected, feedback is suppressed and reform is resisted.

Edgar Schein’s model of organisational culture offers a structured way to explore the disjuncture between the values organisations claim to uphold and the often contradictory behaviours that are embedded in their day-to-day operations.^10^ Schein’s model identifies three internal levels at which culture operates and the visible and invisible dynamics that underpin cultural norms. In addition to these, I also consider how culture is shaped and reinforced across hierarchical and structural levels – from organisational leadership and funding limitations, to bureaucratic inertia and the broader systems of healthcare governance. Here, Foucault’s conceptualisation of power and discourse helps with understanding how knowledge and authority are constructed, with particular attention given to the role of language.^11^ Bringing together Schein’s model of organisational culture and Foucault’s theory of power enables a dual focus: first, on the internal cultural tensions that allow contradictions to persist; and second, on the external power relations that normalise and protect those contradictions through discourse and hierarchy.

Argyris and Schön’s theory of organisational learning offers a pathway for imagining constructive alternatives.^12^ Their work, especially on defensive routines and the role of rhetoric, is valuable in highlighting how organisations often resist learning that would challenge deeply held assumptions. By applying their theory, I identify not only the barriers to meaningful change but also practical strategies for cultivating a culture of greater openness and accountability.

Taken together, these three theories provide a coherent, layered framework for diagnosing the cultural pathologies at play within NHS eating disorder services, and for conceptualising change that is not merely procedural, but cultural and epistemic. The practical recommendations arising from this framework are presented in the penultimate section of this paper.

### Epistemic justice and application of lived experience

This work draws on lived experience as a legitimate and situated form of knowledge.^8^ Personal examples are used reflexively and purposefully to illustrate structural and cultural failures in the healthcare system – as well as its successes – that are difficult to capture through traditional research methodologies alone. I use the lens of epistemic justice – defined by Miranda Fricker as the fairness in how people’s contributions to knowledge are heard, interpreted and valued – to question whose experiences are considered credible and whose are ignored within clinical and policy discourses.^13,14^ Similarly, Kristie Dotson’s work on testimonial injustice (the unfair downgrading of a speaker’s credibility) and hermeneutical injustice (gaps in shared interpretive frameworks that make certain experiences harder to understand) informs my analysis of how epistemic injustice is reproduced and sustained.^15^ These inequities are particularly pertinent for groups marginalised from eating disorder research and treatment settings, such as men, ethnic minorities, LGBTQ+ individuals and neurodivergent people.^16,17^

In line with these principles, I treat lived experience as a form of evidence that reveals how systems operate not just in theory, but in practice, and as a lens through which the disjuncture between policy and reality can be more clearly understood. Lived experience is therefore not presented here as a universal truth, but as a grounded narrative that complements existing empirical research and conceptual frameworks. It can also serve a corrective function, helping to identify blind spots, omissions and misrepresentations within research and policy. Overall, my approach aims to challenge dominant hierarchies of knowledge and demonstrate the value of integrating experiential insight into both critique and theory-building.

## Findings and analysis

Schein’s organisational culture theory identifies three internal levels at which culture operates:^11^

- **artefacts:** observable elements, such as policies, protocols and behaviours;
- **espoused values:** stated principles and ideals, which may diverge significantly from actual practice;
- **underlying assumptions:** deep, often unconscious beliefs that shape institutional behaviour and decision-making.

By analysing the artefacts, espoused values and underlying assumptions embedded in NHS eating disorder services, we can begin to understand how organisations sustain practices that appear misaligned with their stated commitments, and why change remains so elusive.

### Artefacts: policies versus practices

NHS eating disorder services espouse commitments to patient-centred care through artefacts such as clinical guidelines, safeguarding policies and multidisciplinary care structures. They are commissioned to follow evidence-based guidelines for all patients with eating disorders,^4,18^ but the existence of artefacts does not mean they are implemented or implemented equally for all groups.^2,3^ For example, diagnostic criteria and treatment approaches for eating disorders are largely based on research conducted in unrepresentative clinical samples^14^ and therefore may exclude diverse identities and presentations of illness, perpetuating feelings of stigma and misunderstanding.^16,17^

Drawing on my own experience for example, I have been excluded from specialist services for many years as a result of being deemed ‘too medically stable’ based on my weight (a direct quote from a letter rejecting my referral). This has been despite the severity of my presentation meeting diagnostic specifiers for ‘extreme’ bulimia nervosa,^19^ and having significant and risky medical complications such as stomach tears and profound electrolyte imbalances. At other times, I have been labelled ‘too unwell to engage with treatment’ as a result of extreme low weight with anorexia nervosa.

Such poorly evidenced and contradictory reasoning illustrates how artefacts may be used on the basis of institutional convenience rather than clinical need. Incoherent adherence to protocols and opaque bureaucratic processes (including sidelining some artefacts in favour of others) can obscure systemic neglect and deepen mistrust between patients and providers.

### Espoused values: misaligned goals

Espoused values, such as commitments to equity and evidence-based care, can also diverge from the realities of service provision. For example, parity of esteem legislation mandates equal prioritisation of mental and physical healthcare.^20^ Yet, in practice, eating disorder services receive disproportionately limited funding,^2,3,21^ reinforcing the inability of providers to offer a more integrated approach to treating patients who often have co-occurring psychiatric, physical and neurodivergent conditions.^16,22^

Where it occurs, the tokenistic engagement of lived experience reflects an organisational culture in which lived experience is valued rhetorically, but marginalised in practice. Although guidelines increasingly acknowledge the value of patient perspectives,^4,5,18^ the mechanisms for integrating these into decision-making often remain superficial, and evidence is lacking on how such contributions influence outcomes. A simple example from my own experience is how I contributed to writing the Royal College of Psychiatrists’ guidelines on the recognition and management of medical emergencies in eating disorders.^5^ Since their publication in 2022, I’ve had more than 20 emergency hospital admissions with severe risk to my life, yet medical professionals involved in my care have frequently been unaware of the guidelines. From my perspective, this has rendered my involvement in their creation performative rather than impactful.

### Underlying assumptions: structural biases

The most insidious failures in eating disorder services stem from unconscious assumptions that shape behaviour and decision-making. Stigmas surrounding eating disorders often portray them as self-inflicted or trivial conditions^7,8^ – a framing that discourages help-seeking, particularly among groups already marginalised within healthcare systems. Clinical settings frequently replicate these biases. For example, assumptions that eating disorders predominantly affect young, White, cisgender women continue to dominate public health messaging, research participation and clinical practice.^8,16^ These narrow frameworks exclude people with intersecting vulnerabilities, compounding barriers to seeking care.^17^

Unconscious assumptions also influence how dissent is interpreted within services. Patients and caregivers who advocate for alternative treatments or raise concerns have described being pathologised by providers, with their actions framed as symptoms of their illness rather than valid responses to systemic failures.^8,23,24^ Such responses shift the responsibility for poor outcomes onto patients, reinforcing cycles of exclusion and harm.

Diagnostic overshadowing is a particularly acute form of this problem for neurodivergent patients.^17^ As an autistic individual, my requests for greater help communicating with care providers and coordinating my treatment were labelled as me ‘demanding special treatment’, rather than being seen as requests for reasonable adjustments. This example demonstrates Foucault’s emphasis on the role of language in constructing and maintaining discourse, with stigmatising narratives and discourses of ‘untreatability’ having the potential to deflect attention from examining providers’ ability to treat effectively.^23^

### The normalisation of neglect

Taken together, this evidence and critical analysis support a characterisation of adult eating disorder treatment in the UK as a system in which neglect has become normalised in many settings. Only a small fraction of adults with eating disorders receive treatment,^2^ and even those with long-standing and severe illnesses may be left without medical oversight and the intensive, integrated care that they need.^25^ I know from my own experience how the routine denial of healthcare can be profoundly damaging. Being refused help for severe illness over many years – and despite seeking help – has been traumatically invalidating and has actively contributed to feelings of worthlessness, self-blame and severe difficulty regulating my emotions. A lack of effective treatment from sufficiently trained staff has contributed to extreme risk to life, preventable hospital admissions and the diagnostic overshadowing of major co-occurring conditions, which went untreated for decades. This ultimately amplified the burden I placed on an already strained and disjointed healthcare system, where my treatment has rarely been integrated across well-resourced services. Long-standing marginalisation from the care I need has led me to perceive neglect itself as the central feature of my experience living with an eating disorder.

Such emotional and physical harm extends beyond patients, also affecting their families, carers and communities.^26^ Families often witness ongoing neglect, sharing feelings of helplessness as they advocate for their loved ones, only to have their concerns dismissed.^8,23^ This shared sense of impotence fosters mistrust in healthcare systems and strains familial relationships. Communities that lose trust in healthcare institutions are less likely to seek early intervention, worsening long-term outcomes.^27^ Achieving greater equity therefore requires a trauma-informed and restorative approach to care that emphasises repairing relationships, addressing systemic harm and rebuilding trust between patients, caregivers and services.^28^

### Shared trauma: a failing system perpetuates dual harms

Although the iatrogenic harms of eating disorder services are often considered only in relation to patients, the emotional and ethical toll on professionals working within these systems is equally pressing. The same institutional logics that deny or delay care for patients can also render clinicians powerless, creating conditions in which moral injury becomes a daily occupational hazard.^6,28^ Moral injury arises when healthcare workers are unable to provide the care they know is needed because of systemic constraints – be they service thresholds, funding limitations, or rigid protocols that leave no room for individualised judgement.

Clinicians are also caught within the architecture of epistemic injustice. When professional instincts are overridden by inflexible guidance or managerial targets, knowledge derived from clinical experience may be discounted in much the same way that patients’ lived experiences can be, especially where they diverge from established norms. A tension arises between the emphasis placed within evidence-based medicine on treatments that are proven effective, on average, through large-scale studies,^29^ and the clinical reality of patients with more complex or atypical presentations that do not fit neatly into standard protocols.

As a result, clinicians may feel restricted from tailoring care to individual needs, even when such flexibility is clinically indicated for a particular patient.^6^ For instance, several practitioners I have encountered have expressed frustration at not being able to offer more appropriate support, citing service eligibility criteria, waiting list pressures and the lack of sufficiently evidenced treatment options for my presentation. The fear of professional censure or losing a medical license may also play a role in inhibiting compassionate decision-making.

Together, these constraining factors risk creating a climate of mutual trauma. On the one hand, patients experience neglect, invalidation and preventable harm; on the other, providers may experience helplessness, burnout and ethical dissonance. These harms are relational and systemic, not simply individual, and point to the need to reconceptualise iatrogenesis, not as a one-way dynamic inflicted on patients, but as an emergent property of dysfunctional systems that damages everyone involved.^28^ Without addressing this dual experience of harm, interventions aimed at improving eating disorder services are likely to remain superficial. What is needed is a culture shift towards trauma-informed systems that centre relational ethics, emotional safety and co-produced flexibility – offering restoration not just for patients and their caregivers, but also for the staff tasked with caring for them.

### Ways forward: towards a culture of responsibility

When the healthcare system fails to listen, it fails to learn, as valuable insights into patient needs are lost. By obstructing improvement, a culture of ignorance perpetuates cycles of harm and deepening mistrust between patients and providers. Rebuilding trust and fostering a culture that values feedback, transparency and collaboration is therefore essential if the healthcare system is to deliver the patient-centred care it aims for and that individuals with eating disorders deserve. Central to this task is dismantling the status quo and creating something that I term a ‘responsibility culture’.

A responsibility culture is one that prioritises transparency about the limits of treatment provision, and reflexivity across individual, team and organisational levels. Vitally, it must include greater collaboration, centring on the views, preferences and experiences of patients, carers and their communities – ensuring that diverse and marginalised voices are meaningfully included in everything from day-to-day clinical decisions about their care to organisational leadership roles.^8^

Many examples from my personal experience demonstrate that such an approach is both possible and beneficial, even within the constraints of existing specialist services. For instance, when accessing eating disorder treatment in recent years, I encountered differing responses from two services to concerns I raised about a lack of information during the process of referral, assessment and initiating treatment. In one service, the response was defensive, with comments implying I should be grateful for the prospect of being offered any treatment at all. By contrast, the other service invited me to a 90 min meeting where we re-designed patient information and discussed reasonable adjustments to the intake process, which have since been extended to other neurodivergent patients. During this meeting, I never felt blamed for the challenges I had faced; instead, the responsibility for learning how to make the process easier was shared. Similarly, it has been helpful when providers have been transparent regarding their lack of resources. This has helped me to understand that my difficulties accessing care have not arisen from personal failure, lack of worthiness, or having an insufficiently ‘valid’ eating disorder.

Beyond my own experiences and individual clinical encounters, there are positive examples of learning and adaptation where co-production and transparency have led to tangible organisational change. For instance, work has been done in partnership with autistic patients to redesign assessment processes, introduce more flexible engagement pathways and implement neurodivergent-friendly adjustments such as reduced sensory load in in-patient settings – leading to better treatment outcomes.^29^ There is also an emerging body of research that illustrates the beneficial role of co-production for improving wider team culture, reducing staff burnout and improving morale.^30^

Research from other areas of mental health supports this direction. For example, Dickens et al evaluated a mandated ‘culture improvement programme’ within in-patient mental health services in the East of England.^31^ They found small but measurable shifts away from rigid, hierarchical ‘clan’ structures, towards a culture that was more ‘adhocratic’ – meaning characterised by flexibility, innovation and decentralised decision-making. This shift suggests that even in traditionally bureaucratic settings, change towards more adaptive and reflexive cultures is possible, especially when staff at multiple levels are engaged.

Similarly, Repper and Eve describe a case study of an independent mental health consultancy in the UK embedding co-production across every level of its work – from service design to strategic leadership.^32^ Their practical insights highlight the importance of managing power differences, accommodating diverse needs and generating new possibilities through shared ownership, rather than merely selecting from existing, professionally-led solutions. Meanwhile, Eljiz et al present an Australian case study demonstrating how large-scale organisational redesigns can be underpinned by co-production principles.^33^ They note that meaningful patient involvement in these processes enables a shift in values, behaviours and outcomes across the entire healthcare system, illustrating that co-design not only fits with structural change but can actively drive it.

These examples of innovation and adaptation, even within compromised systems, suggest that some of the failures in eating disorder services I have described may be less about the inevitability of systemic dysfunction and more about entrenched cultural norms that can, in fact, be beneficially disrupted. Recognising variations across regions and approaches strengthens the argument for change by illustrating that transformation is not only possible, but is already happening in places where power and responsibility are more equitably shared and organisational flexibility is prioritised. These principles are relevant for all treatment providers and need to be prioritised in the UK context as part of any future reorganisation of the NHS.

### Practical recommendations

Argyris and Schön’s theory of organisational learning offers insights into how organisations either adapt and improve in light of feedback, or persist in practices that reinforce ignorance and inequities,^12^ making this a relevant framework for identifying how the field of eating disorders may move towards a more feedback-responsive culture. Table 1 outlines four key conceptual shifts they describe and how each of these may be applied to achieve meaningful cultural change in the context of eating disorders. Each conceptual shift is provided with a definition, explanation and practical recommendations for its implementation.

**Table 1:** Four conceptual shifts required in eating disorder service culture, based on Argyris and Schön’s theory of organisational learning^12^ Table 1 long description.

Conceptual shift and definition	In the context of eating disorder treatment	Recommendations
1 From single-loop to double-loop learning		
Single-loop learning involves making surface-level adjustments without questioning underlying assumptions, policies or norms. Double-loop learning, by contrast, examines and revises these assumptions to address root causes of systemic issues.	Single-loop learning is evident when organisations focus on superficial corrections such as creating guidelines, without addressing structural inequalities that stall their implementation for all groups.	To move towards double-loop learning, services must interrogate the norms and assumptions that sustain inequities. This includes addressing the lack of training and awareness of good-practice guidelines, and their poor implementation. Greater reflexivity and co-designed feedback mechanisms can help reframe entrenched biases. Partnering with advocacy groups and ensuring resources are allocated to underserved populations can further drive systemic reform.
2 From espoused values to theories-in-use		
Similar to Schein’s perspective, espoused values are the principles and commitments an organisation claims to uphold. Argyris and Schön differentiate these from ‘theories-in-use’ – the actual behaviours and practices that guide decision-making, which often diverge from stated values.	Although services frequently espouse values including equity, inclusivity, valuing feedback and providing integrated care aligned with patients’ rights, they often fail to reflect these in practice.	Bridging the gap between values and practice requires the redistribution of decision-making power and questioning who gets to decide whether values are enacted or not. Embedding lived experience in every stage of service design, delivery and evaluation can help align commitments to equity with tangible outcomes. Proactively seeking feedback and providing lived experience-led training on systemic inequities can further operationalise these values.
3 From defensive routines to reflexivity		
Defensive routines are strategies organisations use to protect themselves from perceived threats such as blame or criticism: they stifle learning and adaptation by deflecting feedback.	Defensive routines manifest in dismissive responses to feedback. These behaviours reinforce existing hierarchies and prevent organisations from reflecting on systemic issues.	Overcoming defensive routines requires normalising reflexivity. Organisations should create spaces for open dialogue between clinicians, patients and caregivers and allocate time for professionals to reflect on their biases and assumptions. Transparent, co-designed feedback and complaints mechanisms can rebuild trust, while trauma-informed restorative practices can address harm caused by past defensiveness.
4 From a rhetoric of learning to a practice of learning		
The rhetoric of learning involves performative gestures that project openness and inclusivity without enacting real change. The practice of learning requires meaningful engagement with feedback to drive systemic reform.	The rhetoric of learning is evident in superficial measures and tokenistic involvement of lived experience, reinforcing the status quo while masking structural inequities.	To move from rhetoric to practice, services must co-design tools, treatments and research with diverse patient groups, amplifying marginalised voices in leadership roles and promoting epistemic justice. Transparent data collection should drive innovation based on the priorities of people with lived experience.

### Conclusions

This paper has analysed the unhelpful cultural dynamics that have been evidenced within NHS eating disorder services and how they operate on different levels to perpetuate systemic failures that have an adverse impact on patients, caregivers and healthcare providers alike. The problems I have highlighted will not be resolved only by attending to pressing resource constraints,^2,21^ creating yet more artefacts of care, or by much-needed efforts to prevent eating disorders arising in the first place. They will also not be resolved by assigning blame or deepening divisions between those who participate in eating disorder care.

Instead, the eating disorder field needs to confront its persistent knowledge and treatment gaps with a culture of shared responsibility. This involves first acknowledging the striking disparity between what is aimed for and what is offered by eating disorder services. Accepting – rather than ignoring – this reality requires resisting superficial solutions, defensive routines and tokenistic rhetoric, instead embracing greater reflexivity, openness and practices of shared learning. This cultural shift will enable the positive values, artefacts and language of eating disorder care to become more of a reality in how that care is experienced by the people who need it.

Alongside many others, I have made repeated calls over many years for the alarms to ‘no longer be ignored’.^2^ Addressing the immediate crises that cause the alarms to sound is not enough: more radical change is needed that addresses both the structural factors giving rise to failures in care and the culture of ignorance that serves to deny them. More than this, treatment provision must be capable of actively redressing the suffering caused by systemic failures, and the experiences of those most harmed need to be held at the heart of creating a more compassionate approach. Only a collaborative approach will be capable of restoring that which has been lost to such failures – be that health, hope, or faith in the system itself.

## About the author

**James Downs**, MEd (Cantab), is an independent researcher and patient based in Cardiff, UK.

## Table 1 Long description

The table has four rows and three columns. The columns are labeled Conceptual shift and definition, In the context of eating disorder treatment, and Recommendations. The rows are labeled 1 From single-loop to double-loop learning, 2 From espoused values to theories-in-use, 3 From defensive routines to reflexivity, and 4 From a rhetoric of learning to a practice of learning. Row 1: Single-loop learning involves making surface-level adjustments without questioning underlying assumptions, policies or norms. Double-loop learning examines and revises these assumptions to address root causes of systemic issues. Single-loop learning is evident when organisations focus on superficial corrections such as creating guidelines, without addressing structural inequalities that stall their implementation for all groups. To move towards double-loop learning, services must interrogate the norms and assumptions that sustain inequities. This includes addressing the lack of training and awareness of good-practice guidelines, and their poor implementation. Greater reflexivity and co-designed feedback mechanisms can help reframe entrenched biases. Partnering with advocacy groups and ensuring resources are allocated to underserved populations can further drive systemic reform. Row 2: Espoused values are the principles and commitments an organisation claims to uphold. Theories-in-use are the actual behaviours and practices that guide decision-making, which often diverge from stated values. Although services frequently espouse values including equity, inclusivity, valuing feedback and providing integrated care aligned with patients rights, they often fail to reflect these in practice. Bridging the gap between values and practice requires the redistribution of decision-making power and questioning who gets to decide whether values are enacted or not. Embedding lived experience in every stage of service design, delivery and evaluation can help align commitments to equity with tangible outcomes. Proactively seeking feedback and providing lived experience-led training on systemic inequities can further operationalise these values. Row 3: Defensive routines are strategies organisations use to protect themselves from perceived threats such as blame or criticism; they stifle learning and adaptation by deflecting feedback. Defensive routines manifest in dismissive responses to feedback. These behaviours reinforce existing hierarchies and prevent organisations from reflecting on systemic issues. Overcoming defensive routines requires normalising reflexivity. Organisations should create spaces for open dialogue between clinicians, patients and caregivers and allocate time for professionals to reflect on their biases and assumptions. Transparent, co-designed feedback and complaints mechanisms can rebuild trust, while trauma-informed restorative practices can address harm caused by past defensiveness. Row 4: The rhetoric of learning involves performative gestures that project openness and inclusivity without enacting real change. The practice of learning requires meaningful engagement with feedback to drive systemic reform. The rhetoric of learning is evident in superficial measures and tokenistic involvement of lived experience, reinforcing the status quo while masking structural inequities. To move from rhetoric to practice, services must co-design tools, treatments and research with diverse patient groups, amplifying marginalised voices in leadership roles and promoting epistemic justice. Transparent data collection should drive innovation based on the priorities of people with lived experience.

Navigate back to Table 1.
